# Pathological replication in cells lacking RecG DNA translocase

**DOI:** 10.1111/j.1365-2958.2009.06773.x

**Published:** 2009-07-01

**Authors:** Christian J Rudolph, Amy L Upton, Lynda Harris, Robert G Lloyd

**Affiliations:** Institute of Genetics, University of Nottingham, Queen's Medical CentreNottingham, NG7 2UH, UK.

## Abstract

Little is known about what happens when forks meet to complete DNA replication in any organism. In this study we present data suggesting that the collision of replication forks is a potential threat to genomic stability. We demonstrate that *Escherichia coli* cells lacking RecG helicase suffer major defects in chromosome replication following UV irradiation, and that this is associated with high levels of DNA synthesis initiated independently of the initiator protein DnaA. This UV-induced stable DNA replication is dependent on PriA helicase and continues long after UV-induced lesions have been excised. We suggest UV irradiation triggers the assembly of new replication forks, leading to multiple fork collisions outside the terminus area. Such collisions may generate branched DNAs that serve to establish further new forks, resulting in uncontrolled DNA amplification. We propose that RecG reduces the likelihood of this pathological cascade being set in motion by reducing initiation of replication at D- and R-loops, and other structures generated as a result of fork collisions. Our results shed light on why replication initiation in bacteria is limited to a single origin and why termination is carefully orchestrated to a single event within a restricted area each cell cycle.

## Introduction

Accurate replication of DNA and faithful transmission of duplicated chromosomes are major challenges for dividing cells, particularly when faced with damage to the DNA. In eukaryotes, a variety of surveillance mechanisms (checkpoints) make sure cells progress through the cell cycle only when appropriate to do so. For instance, the G_1_–S transition checkpoint inhibits initiation of DNA replication if there are lesions in the template. This delay provides time for repair activities to restore the template, after which replication might proceed unhindered. Without such co-ordination, there is increased risk of mutation, genomic instability and cell death (Myung *et al*., 2001). In contrast, there appears to be no G_1_–S checkpoint in bacteria. Replication forks stall at lesions and undergo time-consuming processing before replication restarts, providing an opportunity for repair activities to clear the path ahead. This delay in finishing chromosome replication does not lead to a delay in origin firing and new rounds of replication are initiated even if the chromosome has not been fully replicated (Rudolph *et al*., 2007).

The initiation of replication in *Escherichia coli* requires binding of DnaA protein to the single origin of replication (*oriC*), which causes opening of the duplex and facilitates transfer of DnaB helicase from a DnaB–DnaC complex to each of the template strands. This leads to the assembly of two replisomes, which then move away from *oriC* in opposite directions (Messer, 2002). Duplication of the circular chromosome is achieved when two forks meet in a region opposite the origin flanked by a number of polar *ter* sequences that ensure replication terminates in this region even when the progress of one of the forks is delayed (Neylon *et al*., 2005; Duggin *et al*., 2008). Thus, the chromosome is divided into two replichores and termination is restricted to a specialized area containing additional genetic elements that orchestrate chromosome segregation (Reyes-Lamothe *et al*., 2008).

However, the replisomes meeting in the terminus area may not be the ones starting initially at *oriC*, but new ones assembled along the way. A number of studies have indicated that forks may stall or even collapse as they encounter obstacles in or on the template strands (Michel *et al*., 2007). Exactly what happens when a fork does stall remains far from clear but is likely to vary according to the blocking lesion (McGlynn and Lloyd, 2002a; Michel *et al*., 2007; Rudolph *et al*., 2007). The need to reload DnaB in cells irradiated with UV light suggests that the replisome dissociates, at least partially, in which case a new replisome has to be assembled in order to finish replication (Rudolph *et al*., 2007). Replication restart is mediated in such cases by PriA protein, which targets the fork and facilitates loading of DnaB and subsequent replisome assembly (Heller and Marians, 2006). PriA has a DNA helicase activity that can unwind any nascent lagging strand at the branch point to create a landing pad for DnaB (Heller and Marians, 2006). However, helicase defective PriA proteins retain the ability to promote restart and thus strains expressing these proteins lack the reduced cell viability and high sensitivity to DNA damage associated with *priA* null strains (Jaktaji and Lloyd, 2003). Their viability most probably reflects a compensating activity of one or more of the other helicases (RecG, RecQ, Rep, UvrD) implicated in the remodelling of stalled forks (McGlynn and Lloyd, 2002a; Mahdi *et al*., 2006; Heller and Marians, 2007; Michel *et al*., 2007).

RecG is unusual among the helicases that target forks in that it translocates on duplex DNA and has the ability to interconvert fork and Holliday junction structures, at least *in vitro* (McGlynn and Lloyd, 2000). The very low viability of strains lacking both RecG and PriA, coupled with the fact that the *recG* null phenotype is suppressed by mutations reducing the helicase activity of PriA provides support for the idea that RecG promotes replication *in vivo* (Al-Deib *et al*., 1996; McCool and Sandler, 2001; Gregg *et al*., 2002; Jaktaji and Lloyd, 2003). However, the wide range of DNA substrates that can be unwound by RecG and the pleiotropic phenotype of *recG* mutants make it difficult to pin down exactly what the protein does (McGlynn and Lloyd, 2002a,b;).

DNA damage, beside being a severe threat for ongoing replication, can also cause the formation of new replication forks in other chromosomal areas after induction of the SOS response (Kogoma, 1997). This has been described as inducible stable DNA replication (SDR), referring to the fact that this replication, unlike *oriC*-initiated replication, is resistant to inhibition of protein synthesis and does not require the initiator protein DnaA. Inducible SDR has been shown to be dependent on recombination (Kogoma, 1997) and is exacerbated in the absence of RecG (Hong *et al*., 1995). SDR can also occur constitutively in cells lacking certain proteins, including RecG (Hong *et al*., 1995; Kogoma, 1997).

In this paper we report studies of DNA replication and cell cycle progression in the absence of RecG and the effect UV-induced damage has on these processes. We demonstrate that irradiated *recG* cells suffer significant and persistent defects in chromosome segregation, despite evidence of their having replicated the origin and terminus areas. The observed defects in chromosomal segregation are associated with dramatically increased levels of DnaA-independent DNA synthesis. We show that a mutation inactivating PriA helicase suppresses the segregation defect very effectively and also leads to a dramatic reduction of DnaA-independent replication. Our data suggest that UV-induced damage leads to an increase in the number of replication forks travelling the chromosome and that this pathological replication creates a problem for orderly chromosomal segregation that persists long after the UV-induced lesions have been excised. We propose that RecG decreases the number of replication forks in UV-irradiated cells by limiting UV-induced, DnaA-independent initiation of replication and also reduces the pathological consequences of subsequent uncontrolled chromosomal amplification.

## Results

### Defective division of UV-irradiated *recG* cells

**Fig. 1:**
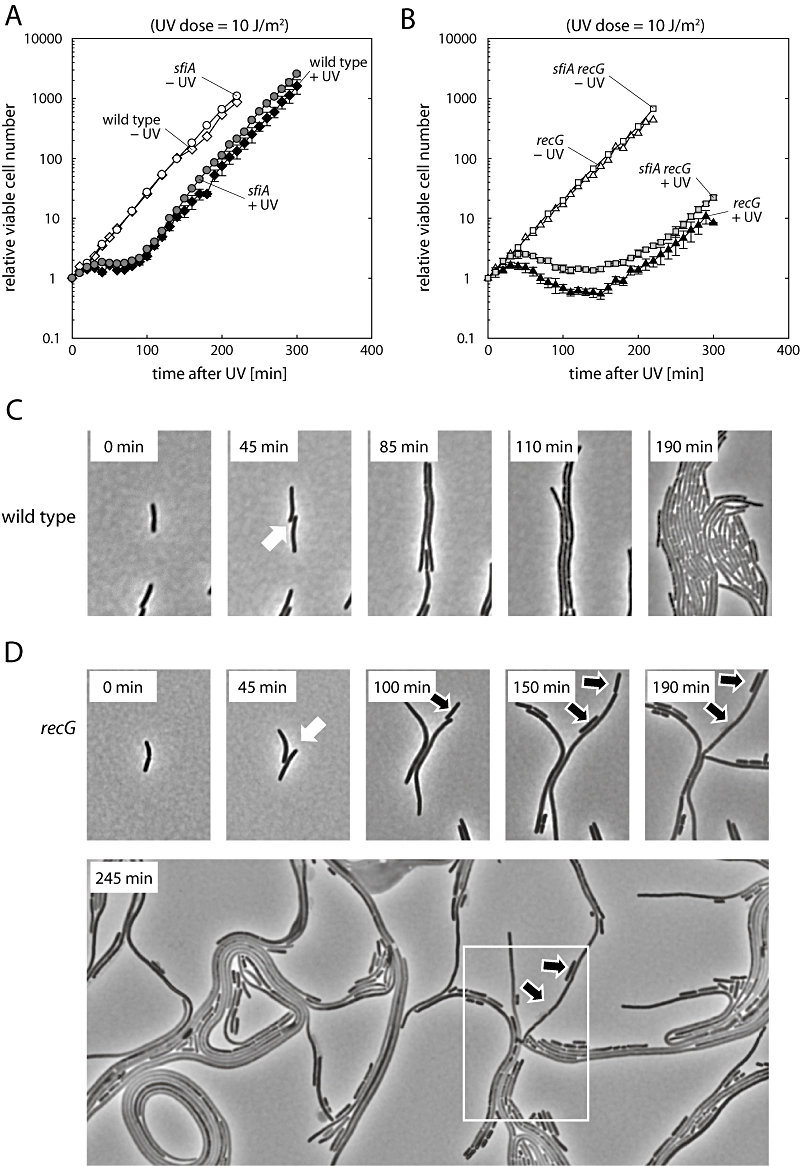
Effect of RecG on cell cycle progression. A and B. Cell replication following UV irradiation. The strains used were MG1655 (wild type), N5209 (*sfiA11),* N4560 (*recG)* and N5225 (*sfiA11 recG*). Data are means (± SE) of three experiments for irradiated and two for unirradiated cells. Data for MG1655 and *sfiA11* were reproduced for comparison from Rudolph *et al*. (2007). C and D. Time lapse photography following growth of single cells after UV irradiation. The strains used were MG1655 (wild type) and N4560 (*recG*). White arrows indicate last divisions after irradiation before cells start to filament. Dark arrows illustrate dead cells budded off *recG* filaments either showing no further divisions or bursting, leaving a ‘ghost’ visible at 190 and 245 min. The 245 min time point shows an expanded field of view with the section shown at 0–190 min outlined.

**Fig. 2:**
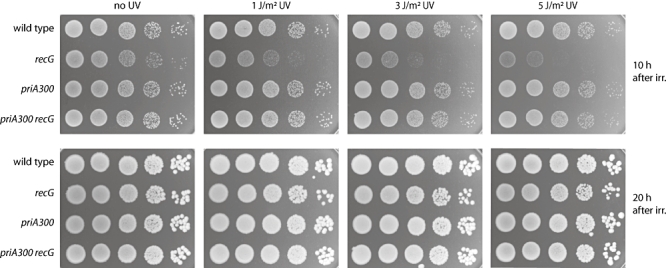
Effect of RecG on viability and cell cycle progression after UV irradiation. Cultures were grown to an A_650_ of 0.48 in LB, diluted in 56/2 and appropriate dilutions spotted onto LB agar. The plates were photographed at the times indicated to illustrate the delay in cell division as well as the differences in viability. The strains used were MG1655 (wild type), N4560 (*recG*), N5500 (*priA300*) and RCe29 (*priA300 recG*).

The irradiation of wild type (*rec*^+^) *E. coli* cells with a modest UV dose resulting in little or no cell death causes cell division to be delayed for 60–70 min (Fig. 1A), regardless of the SOS-induced division inhibitor encoded by *sfiA*, as reported (Rudolph *et al*., 2007). By comparison, division is delayed for much longer (120–150 min) in cells lacking RecG (Fig. 1B). This extended delay is also independent of *sfiA* (Fig. 1B). It is reflected in the slower development of visible colonies of surviving cells, which is apparent even at a dose of 1 J m^−2^ introducing about 40 pyrimidine dimers per chromosome (Fig. 2). Most (> 90%) of the irradiated *recG* cells give rise to colonies of survivors at this dose.

We followed the growth of irradiated single cells using time-lapse microscopy. The majority of wild type cells undergo one division before starting to filament (Fig. 1C, white arrow). Between 60 and 70 min the filaments start to bud off small cells that are viable, as evident from their growth and division. About 190 min after irradiation the filaments have divided down to small growing cells identical to those in unirradiated samples, indicating full recovery. In doing so, most if not all the biomass generated during filamentation is conserved.

**Fig. 3:**
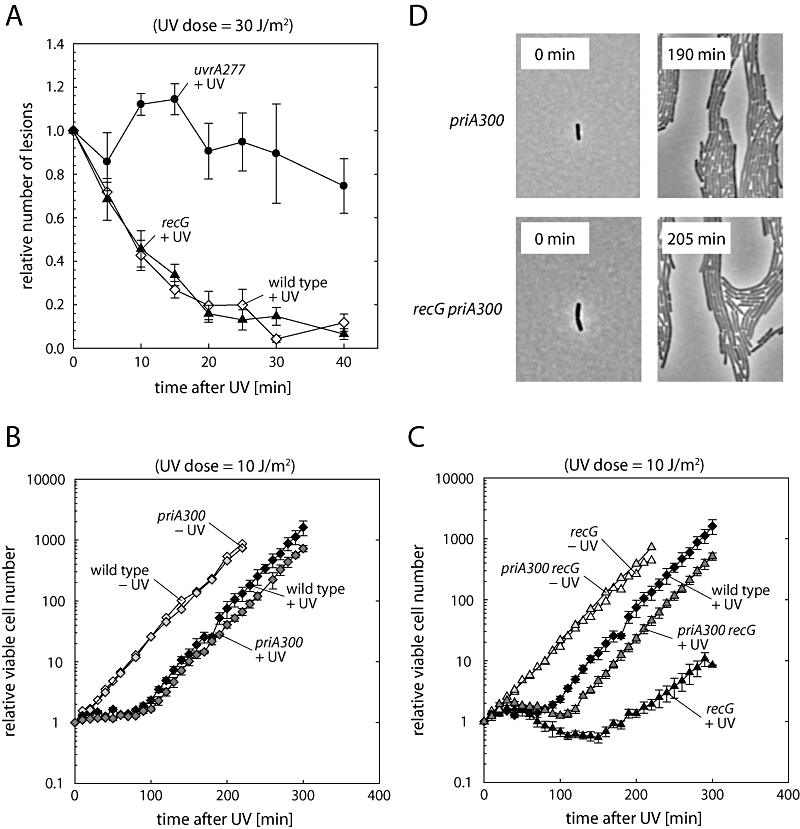
Effect of RecG on cell replication and lesion repair. A. Rate of pyrimidine dimer removal from strain N4560 (*recG*). Data are the mean (± SE) of four independent experiments. Data for MG1655 (wild type) and N4280 (*uvrA*) are reproduced for comparison from Rudolph *et al*. (2007). B and C. Suppression of the division delay in irradiated *recG* cells by *priA300*. Data are means (± SE) of three experiments for irradiated and two for unirradiated cells. The strains used were N5500 (*priA300*) and RCe29 (*priA300 recG*). Data for wild type (MG1655) and *recG* (N4560) were reproduced from Fig. 1 for comparison. D. Time lapse photography following growth of single cells after UV irradiation. The strains used were N5500 (*priA300*) and RCe29 (*priA300 recG*).

A similar initial filamentation is observed with a *recG* mutant, with small cells budding off at both ends between 60 and 70 min after irradiation. However, in contrast to the wild type, these early budded cells are inviable. Some divide once or twice, forming minicells capable of no further growth (Fig. 1D). At later times, most of the *recG* filaments bud off small cells, which either grow to form further filaments or divide like unirradiated cells, consistent with recovery. But complete breakdown (division) of filaments is observed only very rarely. These features are clearly illustrated in Movies S1 and S2. Thus in contrast to the wild type, the bulk of the *recG* cell biomass generated following irradiation persists as very long filaments (Fig. 1D and 245 min time point), thus explaining the delayed appearance of colonies of survivors (Fig. 2). These colonies arise from the small cells budded off at later times. As pyrimidine dimers are removed at about the same rate as in the wild type (Fig. 3A), the extended delay cannot be explained by lesions remaining in the DNA and thus delaying chromosome replication.

### PriA helicase is responsible for the extended delay

The sensitivity of *recG* cells to genotoxic agents is suppressed by mutations reducing the helicase activity of the PriA (Al-Deib *et al*., 1996; Jaktaji and Lloyd, 2003). We investigated whether such a mutation could alleviate the division defect in irradiated *recG* cells using *priA300*, an allele encoding a helicase-deficient PriA protein (Heller and Marians, 2006). The presence of this allele reduces the division delay quite dramatically in the case of a *recG* strain but has little effect on replication of the wild type (Fig. 3B and C). Time-lapse microscopy revealed that there remains a somewhat extended period of filamentation in the *priA300 recG* cells but also that filaments subsequently break down rapidly (Fig. 3D and data not shown). Thus, it appears that the helicase activity of PriA is somehow responsible for the delayed division of irradiated *recG* cells.

**Fig. 4:**
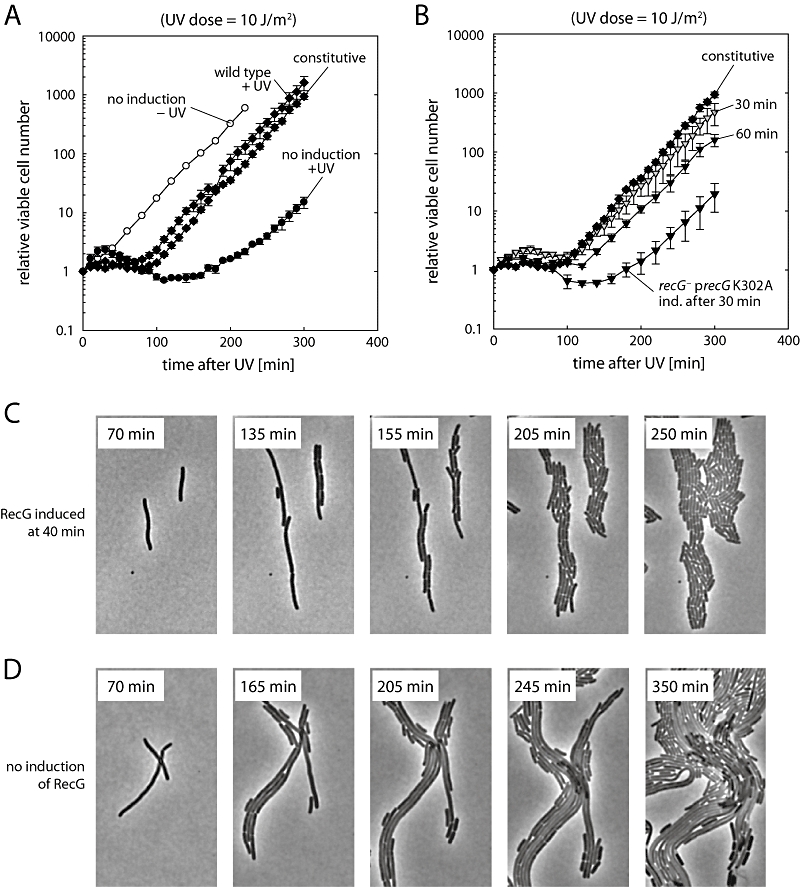
Alleviation of the division delay in irradiated *recG* cells by late RecG expression. A. The division delay of *recG* cells was suppressed by constitutive RecG expression, using strain N4560 (*recG*) harbouring pAM208. N4560 (*recG*) carrying pDIM104 and grown without arabinose (labelled as ‘no induction’) was used as a no RecG control. Data for wild type without plasmid are reproduced from Fig. 1A as a marker. B. For late onset of RecG expression strain N4560 (*recG*) harbouring pDIM104 was used, allowing arabinose-controlled expression of wild type RecG 30 or 60 min after UV irradiation. For late onset expression of the helicase-deficient RecGK302A 30 min after UV irradiation N4560 (*recG*) was used, harbouring pECR12. Data are means (± SE) of three independent experiments. C. Time lapse microscopy of phase contrast images showing filamentation and cellular division of strain N4560 (*recG*) harbouring pDIM104 (*recG*^+^), allowing arabinose-controlled expression of wild type RecG. Expression was induced 40 min after irradiation and the cells followed under the microscope after a 30 min expression period. D. Filamentation of strain N4560 (*recG*) harbouring pDIM104 (*recG*^+^) without induction of RecG expression.

To investigate how PriA helicase might achieve this effect we transformed *recG* cells with a plasmid carrying *recG*^+^ under control of the P_*araBAD*_ promoter. Without arabinose to induce *recG* expression, irradiated cells show a delay in division identical to that in plasmid-free cells, whereas constitutive expression of *recG* results in a pattern very similar to wild type (Fig. 4A). Induction of RecG 30 min or even 60 min after the irradiation is sufficient to shorten the delay significantly (Fig. 4B). Time-lapse microscopy revealed very rapid and complete break down of filaments even if expression of RecG is not switched on until 40 min after UV irradiation (Fig. 4C and D). This effect requires RecG translocase activity as no shortening of the delay is seen with a plasmid encoding RecGK302A (Fig. 4B), which lacks ATPase and DNA unwinding activity (McGlynn and Lloyd, 2001). These experiments suggest that PriA helicase is responsible for the formation or persistence of some stable and toxic DNA intermediate that can be eliminated by subsequent expression of RecG. The fact that this intermediate persists for a long time despite the presence of RuvABC suggests it is unlikely to be a Holliday junction intermediate.

### Chromosome replication and segregation

**Fig. 5:**
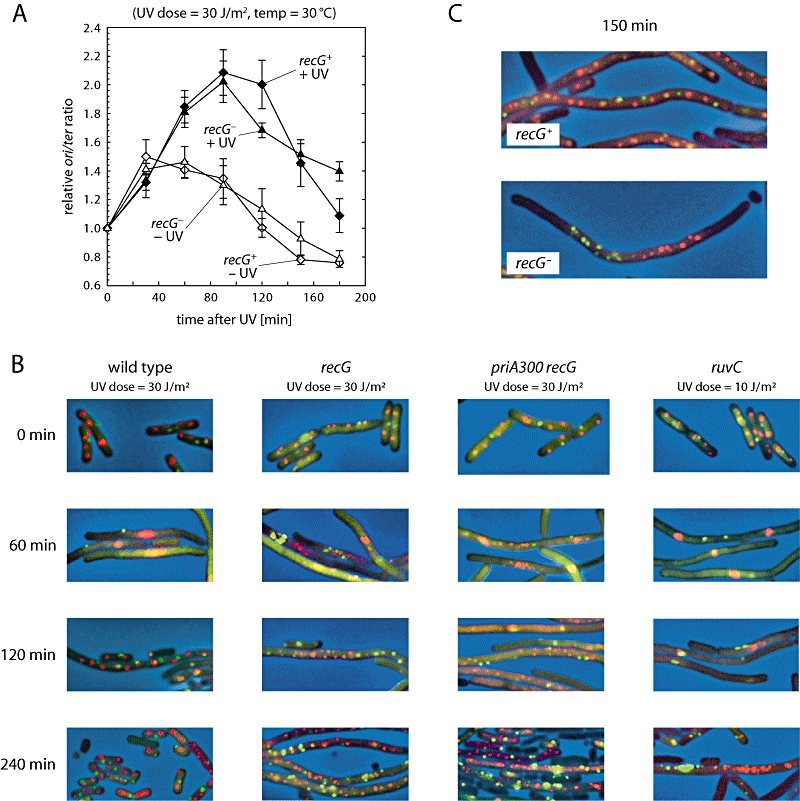
Effect of RecG on replication and segregation of origin and terminus areas. A. Changes in the origin to terminus ratio following irradiation of *recG*^–^ cells. The strain used was RCe111 (*dnaC7 recG*^–^). Cells grown at 30°C were synchronized by incubation at 42°C for 45 min before irradiation and shifting back to 30°C. Data are the mean (± SE) of three independent experiments. Data for RCe79 (*dnaC7 recG*^+^) are reproduced for comparison from Rudolph *et al*. (2007). B. Fluorescence microscopy showing replication and segregation of origin (red foci) and terminus (green foci) areas of the chromosome after UV (combined phase contrast and fluorescence images are shown). The strains used were RCe72 (*recG*), RCe109 (*priA300 recG*) and RCe97 (*ruvC*). Data for the wild type strain (APS345) are reproduced for comparison from Rudolph *et al*. (2007). C. Enlargements of filaments from a repeat of the experiment in B. Data for wild type (APS345) are reproduced for comparison from Rudolph *et al*. (2007).

To investigate what is happening to chromosome replication, we measured the origin to terminus ratio, using cells synchronized before irradiation via a temperature-sensitive *dnaC7* allele. The ratio in *recG* cells mirrored that in wild type cells, with and without UV, and increased in the manner expected for cells able to initiate DNA synthesis after synchronization (Fig. 5A). Greater amplification of the origin after irradiation reflects the continued firing of the origin at times when the terminus has not been replicated because of fork stalling (Rudolph *et al*., 2007).

The similar ability to replicate origin and terminus regions in wild type and *recG* cells evident from the data in Fig. 5A was confirmed by fluorescence microscopy. For this we used strains in which origin and terminus areas of the chromosome were tagged with *lacO* and *tetO* arrays, respectively. The strains carried a plasmid encoding LacI-eCFP and TetR-eYFP repressors to decorate these arrays (Lau *et al*., 2003; Rudolph *et al*., 2007). A strong increase in the intensity of the origin signal was detectable in both wild type and *recG* cells 60 min after irradiation (Fig. 5B; for additional time points see Fig. S1), presumably reflecting the ability of *oriC* to continue firing (Rudolph *et al*., 2007). There is also clear evidence of rapid multiplication and separation of origin foci 90 min after irradiation (Fig. S1). In both cases the number of terminus foci remains low until between 120 and 150 min when they start to multiply rapidly and segregate (Fig. 5B). However, significant differences between wild type and *recG* cells were observed in the patterns of segregation of the replicated origin and terminus regions. In irradiated wild type cells, fluorescent foci corresponding to the replicated origin and terminus areas become regularly interspersed along the cell filaments just prior to the time when most filaments divide down to normal-sized cells (Fig. 5B and C; see Fig. S1 for more extensive images and additional time points). In the case of irradiated *recG* cells, the distribution of foci is less organized, revealing aberrant nucleoid segregation. A different and persistent class of filaments appears that show clusters of origin and terminus foci (Fig. 5B and C). This disorganization makes it impossible to quantify the data. There is also a significant fraction of short cells that show no fluorescence signal, consistent with being anucleate (Fig. S1) (Ishioka *et al*., 1997). However, there are also cells with foci distributed as in unirradiated controls. Their presence is consistent with the viable cells seen to bud from the filaments by time-lapse microscopy.

In *recG priA300* cells, the delay in chromosome segregation is reduced but not entirely eliminated (Fig. 5B). This suggests that DNA intermediates capable of delaying segregation still accumulate, which is in line with the data in Fig. 3B and C. Previous studies showed that *priA300* increases the sensitivity of *ruv* mutants to UV light, indicating that the lack of PriA helicase activity forces cells to rely more on recombination (Gregg *et al*., 2002; Jaktaji and Lloyd, 2003). Indeed, a possible explanation for chromosome clustering in irradiated cells (Fig. 5B and C) would be the accumulation of unresolved recombination intermediates (Ishioka *et al*., 1997). Therefore, we examined irradiated cells lacking RuvABC, which would have difficulty processing such intermediates. As *ruv* cells are more sensitive to UV, we reduced the UV dose to 10 J m^−2^, allowing a level of survival comparable to that of a *recG* mutant at a dose of 30 J m^−2^ (∼20%; data not shown). We observed intense clustering of multiplied origin and terminus foci in these cells and a failure to resolve these clusters even after 240 min (Fig. 5B), consistent with previous studies demonstrating chromosome segregation defects in *ruv* mutants (Otsuji *et al*., 1974; Ishioka *et al*., 1998). However, this does not necessarily mean that the chromosomal segregation defects and division delay observed in *recG* cells are due to the same pathology. We reiterate the fact that RuvABC is available to resolve Holliday junctions.

These studies confirmed that *recG* cells have significant defects in chromosome segregation (Fig. 5B and C), as reported (Ishioka *et al*., 1997). However, they also provided new insight into the reason for this defect.

### Elevation of SDR in *recG* cells

**Fig. 6:**
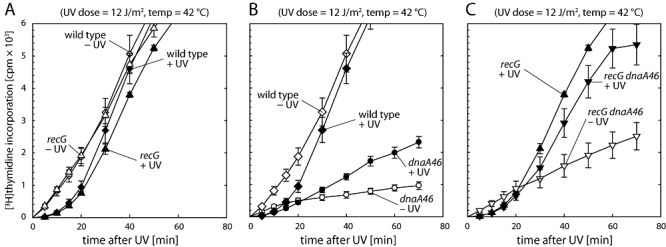
DNA synthesis in *recG* and *dnaA46 recG* cells. A–C. [^3^H]thymidine incorporation in wild type (N1141), *recG* (AU1106), *dnaA46* (AU1068) and *recG dnaA46* (AU1090) cells. Data are means (± SE) of three or more experiments. Data for wild type (N1141) and *dnaA46* (AU1068) are reproduced for comparison from Rudolph *et al*. (2007).

The data presented so far confirm that the *recG* mutant phenotype is efficiently suppressed by mutations inactivating or reducing the helicase activity of PriA. Together with the finding that strains lacking both RecG and PriA have an extremely low viability, these results are strong indicators that RecG might be involved in restart of stalled replication forks (Al-Deib *et al*., 1996; McCool and Sandler, 2001; Gregg *et al*., 2002; Jaktaji and Lloyd, 2003). To investigate DNA replication in further detail, we used [^3^H]thymidine incorporation assays. As shown previously by Donaldson *et al*. (2004), assays of net [^3^H]thymidine incorporation revealed no obvious difference in DNA synthesis between wild type and *recG* cells (Fig. 6A). However, we have shown recently that three processes contribute significantly to thymidine incorporation after UV irradiation: DnaA-dependent initiation of replication at the normal origin of replication (*oriC*), DnaA-independent initiation of SDR at other positions in the chromosome and, thirdly, replication associated with rescue of the forks initially stalled at lesions (Rudolph *et al*., 2007; 2008). Hence, measurements of net [^3^H]thymidine incorporation could be misleading as a delay in one of the three processes might be masked entirely by an increased activity of one of the other two.

When we examined replication in a strain carrying the thermosensitive *dnaA46* allele, we found that *recG* has a quite dramatic effect. In mock-irradiated *dnaA46* single mutants shifted to 42°C, a temperature at which the mutant DnaA protein is inactive, incorporation continues for some time before reducing severely (Fig. 6B), consistent with synthesis by the majority of existing replication forks coming to an end and the failure to initiate new rounds from *oriC* in the absence of DnaA. UV irradiation increases the level of incorporation quite significantly (Fig. 6B) (Rudolph *et al*., 2007). This increase is consistent with induction of SDR, which is defined by its independence of DnaA (Kogoma, 1997).

In mock-irradiated *dnaA46 recG* cells the level of incorporation after the shift to 42°C is already significantly higher than in mock-irradiated *dnaA46* cells (Fig. 6C). This is in line with previous studies reporting increased levels of SDR in *recG* cells (Hong *et al*., 1995). UV irradiation results in a much higher level of incorporation in *dnaA46 recG* cells, almost as high as in a *recG* single mutant (Fig. 6C). Thus, replication is clearly much affected in the absence of RecG, both with and without UV irradiation, a fact that has been missed by the analysis of net [^3^H]thymidine incorporation assays. The very high level of SDR in irradiated cells may create problems for the completion of chromosomal replication. SDR has been suggested to be bidirectional (Kogoma, 1997). Therefore, the continued firing of *oriC* together with initiation of SDR at other sites might result in a drastic increase in the number of replication forks travelling along the chromosome.

### UV-induced synthesis in *recG* mutants is caused by PriA helicase activity

**Fig. 7:**
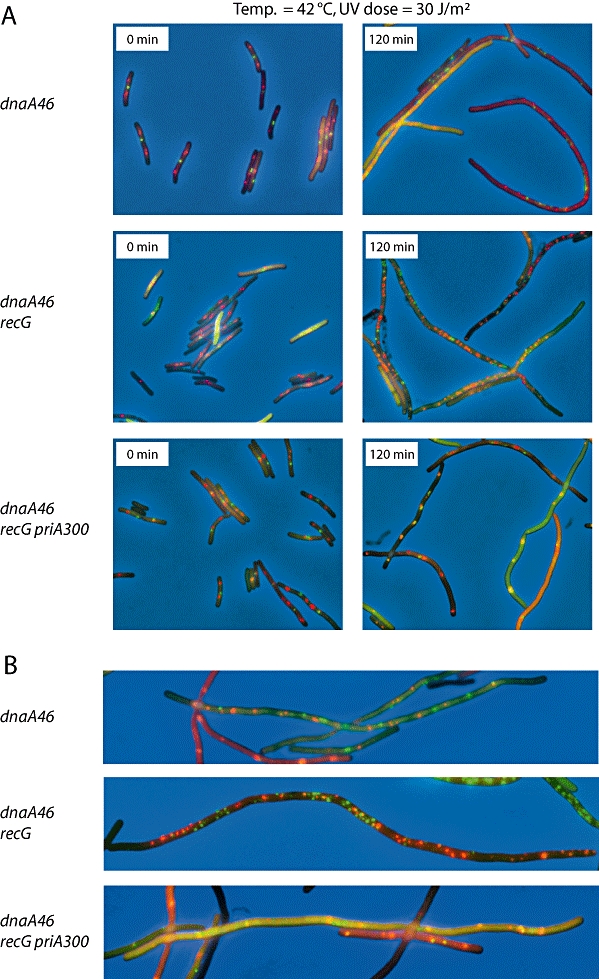
Visualization of UV-induced DnaA-independent synthesis. A. UV irradiation leads to a PriA helicase-dependent increase in origin (red) and terminus foci (green) in the absence of *oriC* firing (combined phase contrast and fluorescence images are shown). Cells were grown at permissive temperature prior to UV irradiation and shifted to 42°C directly after UV. The strains used were RCe197 (*dnaA46*), RCe198 (*dnaA46 recG*) and RCe259 (*dnaA46 recG priA300*). B. Enlargements of filaments from a repeat of the experiment in A.

As [^3^H]thymidine incorporation does not provide any indication of where the SDR is taking place, we investigated chromosomal replication and segregation in *dnaA46* derivatives of strains in which the origin and terminus areas were tagged with fluorescent proteins. The cultures were grown at 30°C, irradiated or mock-irradiated and then shifted to 42°C in order to prevent origin firing. Before the shift to 42°C most *dnaA* cells show multiple origin foci typical for logarithmically growing cells (cf. Figs 5B and 7A). Without UV irradiation, a shift to 42°C leads to domination of the culture with cells showing a single origin and terminus focus within 60–120 min (Fig. S2A). This shows not only that active replication forks finish replication, as suggested by our [^3^H]thymidine incorporation data, but also that in the absence of UV the replicated chromosomes are segregated, enabling cells to divide.

Following UV irradiation, *dnaA* single mutant cells filamented extensively and exhibited a substantial accumulation of origin as well as terminus foci over time (Fig. 7A and B). This accumulation continued over 4 h (data not shown), in line with reports showing that SDR can continue for as long as 20 h (Kogoma, 1997). The origin and terminus foci appear to be regularly interspersed, at least in some of the filaments. Cells showing only a single origin and terminus focus, as observed without UV irradiation, are observed very rarely.

Eliminating RecG from *dnaA* cells had a dramatic effect. UV irradiation leads to a rapid and very dramatic multiplication of both origin and terminus foci (Fig. 7A and B), consistent with the observed increase in SDR. Furthermore, in contrast to the regularly interspersed origin and terminus foci observed in irradiated *dnaA* (*recG*^+^) cells, the amplified origin and terminus foci observed in *dnaA recG* cells tend to form extensive and frequently discrete clusters within the filaments, making quantification of the data impossible.

In order to confirm that active replication is responsible for the observed amplification of the origin and terminus foci, the experiment was repeated in a *dnaC7* temperature-sensitive background. DnaC binds DnaB and is necessary for loading DnaB both during replication initiation at *oriC* and during rescue of stalled forks by PriA/PriC (Marians, 2004). We have also shown that DnaC is necessary for SDR in UV-irradiated cells (Rudolph *et al*., 2007). Following the dynamics of origin and terminus foci in UV-irradiated *dnaC recG* cells revealed that amplification of foci was much reduced (Fig. S2B), confirming that DnaB loading is necessary for amplification of the tagged chromosomal areas, and that this amplification is therefore due to replication.

The extensive amplification of foci in UV-irradiated *dnaA recG* cells is very much in line with our [^3^H]thymidine incorporation data and suggests that, in the absence of RecG, UV irradiation leads to an increased number of replication forks traversing the chromosome. The extreme phenotype led us to speculate that uncontrolled amplification of the chromosome, or of at least certain chromosomal areas, might be responsible for the observed segregation defect and the extreme filamentation phenotype. Our data indicate that, without RecG, PriA helicase is responsible for either the formation or the persistence of some DNA intermediates that result in this pathological replication. We therefore investigated whether SDR is affected in a *recG* background by a helicase-deficient PriA. Given that PriA is involved in loading of the replisome specifically at positions other than *oriC* (Heller and Marians, 2006), a PriA helicase-deficient mutant might show a reduction in SDR. This was exactly what we observed. Following UV irradiation of *dnaA recG priA300* cells, amplification of the origin and terminus was reduced to the level of the *dnaA* single mutant if not lower (Fig. 7A and B). Together these results provide a strong indication suggesting that much of the pathology observed in *recG* cells may actually be due to the increased SDR, which is further amplified by UV-induced damage and continues long after the primary lesions have been repaired. In the absence of PriA helicase activity, SDR is very much reduced, therefore limiting the pathology caused by the lack of RecG.

## Discussion

A functional overlap between RecG and the RuvABC Holliday junction resolvase has been offered previously as an explanation for the modest sensitivity of *recG* cells to UV light (Lloyd, 1991; Lloyd and Sharples, 1993). The results presented here demonstrate that the relatively high survival of UV-irradiated *recG* cells registered in colony assays is deceptive. It masks major defects in cell cycle progression. Thus, most of the biomass generated after irradiation is in the form of extended filaments that persist for a long time and which have problems with chromosome segregation despite having replicated both origin and terminus areas (Figs 1 and 5). Colonies of survivors grow from small cells that bud from these filaments after a considerable delay, leaving behind a morass of filaments full of newly replicated DNA (Figs 1D and 5B). Wild type cells also filament, but less extensively and soon divide down to small cells that grow and divide normally, recovering most of the biomass (cf. Movies S1 and S2).

The extensive filamentation and nucleoid segregation defects recapitulate earlier studies of UV-irradiated *recG* cells that attributed the phenotype to an accumulation of Holliday junctions generated during recombinational repair of daughter strand gaps (Ishioka *et al*., 1997). However, our extended analysis of this phenotype and of how it can be overcome led us to question this explanation. The physical linkage of daughter chromosomes via Holliday junctions may well contribute to the delay of 60 min or so before irradiated wild type cells are able to resume division. It is almost certainly a major reason for the extreme filamentation and poor survival of irradiated cells lacking RuvABC (Fig. 5B) and for the accumulation of branched DNA species in these cells (Ishioka *et al*., 1998; Donaldson *et al*., 2006). However, it is difficult to reconcile with the much-extended delay in division of irradiated *recG* cells. While the branched DNA species observed in *ruv* cells by Courcelle and co-workers persist for a long time, those detected in *recG* cells disappear with the same kinetics as those detected in wild-type cells (Donaldson *et al*., 2006), and presumably for the same reason, i.e. the action of RuvABC. Furthermore the division delay we observed in UV-irradiated *recG* cells is efficiently suppressed by *priA300* (Fig. 3), suggesting that PriA helicase is responsible for either the formation or persistence of some DNA species that is causing segregation defects. This beneficial effect of removing PriA helicase activity is specific to *recG* cells. A *ruv priA300* double mutant shows an exacerbated phenotype in being much more sensitive to UV or mitomycin C than a *ruv* single mutant (Jaktaji and Lloyd, 2003).

Measures of net incorporation of [^3^H]thymidine revealed that wild type and *recG* cells synthesize DNA at comparable rates. There are also similar delays in synthesis following UV irradiation (Fig. 6A), as reported (Donaldson *et al*., 2004). However, our measures of net [^3^H]thymidine incorporation in a *dnaA46* background at 42°C, a temperature at which the mutant DnaA protein is inactive and normal initiation of replication at *oriC* is prevented, revealed that 70–80% of the DNA synthesis in UV-irradiated *recG* cells might be attributable to SDR (Figs 6C and 7). The fraction of the total synthesis attributable to SDR is very much less in UV-irradiated *dnaA46* cells, especially at later time points (Fig. 6B).

By monitoring origin and terminus areas of the chromosome using fluorescence microscopy, we have shown that UV induces extensive initiation of replication in the absence of the DnaA initiator protein, consistent with induction of SDR. More significantly, we found that eliminating RecG has the effect of dramatically increasing amplification of both origin and terminus areas. Furthermore, without RecG the amplified foci observed were frequently clustered in discrete areas of the filamented cells, suggesting a difficulty in chromosome segregation, as if continuing synthesis in the absence of RecG might not be productive in terms of generating fully replicated chromosomes. Although the origin and terminus areas of the chromosomes are clearly being replicated (Figs 5B and 7), it does not necessarily follow that this replication reflects progression of forks from *oriC* to *ter*. Thus, although the overall rate of synthesis is not reduced in *recG* mutants, much of this synthesis appears to be pathological. These studies reinforce the view that net incorporation of radiolabel provides a poor measure of replication fork progression in UV-irradiated cells (Rudolph *et al*., 2007).

Taken together, our results suggest that the prolonged delay in division of UV-irradiated *recG* cells might reflect some debilitating consequence of new replication initiated at sites remote from *oriC*. This hypothesis is further supported by our results with *priA300* strains (Fig. 7). Tanaka and coworkers presented evidence showing that a PriA K230D helicase deficient strain shows a significant reduction of SDR in comparison with wild type strains (Tanaka *et al*., 2003). Our results show that the massive amplification of origin and terminus areas was dramatically reduced in a *recG priA300 dnaA* background (Fig. 7). The suppression of the *recG* filamentation phenotype by *priA300* is therefore associated with a dramatic reduction of SDR, indicating that the efficient suppression of the *recG* mutant phenotype is actually caused by a reduction of SDR.

The precise nature of SDR is still poorly understood and it is even less clear where and how it initiates. There is some evidence of initiation in the origin region and near *ter* (Horiuchi *et al*., 1994; Kogoma, 1997), which might account for some of the multiplication of these areas of the chromosome observed by fluorescence microscopy (Fig. 7). However, in order to achieve the dramatic increase in the number of foci, the areas under investigation need to be replicated multiple times. In theory this could be achieved by multiple and fairly rapid initiation events at defined SDR origins. But, it has been shown that over-initiation at defined origins results in replication fork collapse, because secondary forks are capable of catching up with primary forks, resulting in the formation of linear DNA fragments due to replication run-off (Simmons *et al*., 2004). The same rationale should apply to rapidly firing SDR origins, making it unlikely that this would be the only cause of the rather drastic multiplication of chromosomal areas observed. Furthermore, although the initiation of SDR is induced by UV-induced damage, it continues long after all detectable lesions have been removed (cf. Fig. 3A with Figs 6 and 7). It is not obvious why firing of specific SDR origins should occur once the initial trigger is gone.

We suggest instead that following UV irradiation, the increased number of replication forks travelling the chromosome might itself create a problem. Normally, replication fork complexes travel around the chromosome in a specific orientation and meet in a well-defined termination area. The multiple genetic elements in this area orchestrating termination and chromosome segregation point to the importance of limiting fork encounters to this area (Sherratt, 2003; Bigot *et al*., 2005; Neylon *et al*., 2005; Duggin *et al*., 2008). If all fork encounters allowed nascent strands to be ligated, there would be no obvious reason why unscheduled encounters elsewhere should pose any great difficulty. But the terminus area is a strong hot spot for RecBCD-dependent recombination (Horiuchi *et al*., 1994), indicating perhaps that termination does not always proceed smoothly, sometimes resulting in the formation of duplex DNA ends. Indeed, studies of DNA replication *in vitro* revealed that without Tus to curb fork movement, a replisome may displace the 3′ end of the nascent leading strand made by the fork coming in the other direction, generating a branched DNA structure that allows re-replication of the already replicated DNA (Hiasa and Marians, 1994). Such events might account for the over-replication observed in the absence of a functional termination system (Krabbe *et al*., 1997; Markovitz, 2005).

In wild type cells, UV irradiation induces formation of new replication forks. Presumably such forks could continue to replicate the chromosome until they merged with forks coming from *oriC*. If the additional fork encounters increase the likelihood of some hiccup, such as the displacement of the nascent 3′ strand by the DnaB helicase of the converging fork, the 3′ flap structure might be modified by RecG translocase or eliminated by one of the several 3′ exonucleases in *E. coli*, thus limiting the use of the flap to assemble additional forks at this site. RecG is particularly well suited to carry out such modification (McGlynn and Lloyd, 2001; Singleton *et al*., 2001). In the absence of RecG, some flaps might persist despite the presence of 3′ exonucleases and thus provide targets for PriA, leading to the assembly of new replisomes that might re-replicate parts of the chromosome, leading to a partial increase of some chromosomal areas. As partially replicated chromosomes cannot be segregated, this scenario would explain the clustering of foci observed in the fluorescence microscopy images (Figs 5 and 7).

Thus, the role of RecG would be twofold. First its presence would eliminate some of the D- and/or R-loops that prime SDR and second it would limit the opportunity for PriA to re-initiate replication following unscheduled encounters between converging forks, thus reducing pathological replication and allowing earlier cell division. In this model, over-replication by SDR would lead to an ongoing formation of 3′ flaps, providing an explanation for the observation that SDR can continue for many hours (Kogoma, 1997). The observed rescue of UV-irradiated *recG* cells by late expression of RecG from a plasmid might therefore reflect that ability of RecG to remove the substrate causing the over-replication. This would allow complete replication of the multiple copies of the chromosome seen in filamentous cells, leading to the rapid chromosome segregation and break down of the filaments observed subsequently (Fig. 4).

This model does not rule out the idea that RecG can help to resolve Holliday junctions. However, it does raise the possibility that any recombination reaction leading to additional replication forks may have pathological consequences in cells lacking RecG that reduce viability and hence the ability to recover recombinant products.

The model outlined fits very well with the replichore arrangement of the bacterial chromosome. By reducing fork collision to a single event per cell cycle, the risk of re-replication of the already replicated DNA is minimized. By orchestrating collision within a defined area, complete replication of the chromosome can be registered, which may explain why additional sequences have evolved within this region to direct FtsK-mediated chromosome segregation during cell division (Reyes-Lamothe *et al*., 2008). The induction of SDR compromises these advantages, and is also likely to increase the likelihood in head on collisions between DNA and RNA polymerase complexes, which may go some way towards explaining why *ter* sites flank some 40% of the *E. coli* chromosome.

## Experimental procedures

### Bacterial strains and plasmids

All strains are derivatives of *E. coli* K-12 (Table 1). For fluorescence microscopy, strains carrying *lacO240* and *tetO240* arrays were transformed with pLAU53, which encodes arabinose-inducible LacI-eCFP (enhanced cyan fluorescent protein) and TetR-eYFP (enhanced yellow fluorescent protein) (Lau *et al*., 2003). pDIM104 carries the *recG* coding sequence amplified with primers 5′-cgcatgccatggaaggtcgcctgttagatgctgtc and 3′-cgcatgctcgagtgcagacgcattcgagtaacgttccg, cloned behind the P_*araBAD*_ promoter of pLAU17 (Lau *et al*., 2003) using the NcoI and XbaI sites, thus replacing eCFP. For expression of a helicase-deficient *recG*K302A gene the same steps were applied except that plasmid pAM219 (Mahdi *et al*., 2003) was used for amplification of *recG*K302A, generating pECR12. pAM208 was as described (Mahdi *et al*., 2003).

**Table 1:** *Escherichia coli* K-12 strains.

Strain	Relevant genotype^a^	Source
General P1 donors		
BW6164	*thr-43::*Tn*10*	CGSC
JJC395	*sfiA11*	Bénédicte Michel (Dri *et al*., 1991)
JJC396	*sfiA100::kan pyrD*	Bénédicte Michel
N3793	Δ*recG263::kan*	Al-Deib *et al*. (1996)
N4452	Δ*recG265::cat*	Jaktaji and Lloyd (2003)
NY171	*deo-41 dnaC7*	CGSC
RUC663	*tnaA::*Tn*10 dnaA46*	Tove Atlung
N1141 and derivatives^b^		
N1141	F^–^*lacI3 lacZ118 metE70 leuB6 proC32 thyA54 deo(BC) malA38(?)araC14 mtl-1 xyl-5 rpsL109 rpsE2015 gyrA265 supD*	Rudolph *et al*. (2007)
AU1068	*tnaA::*Tn10 *dnaA46*(ts)	Rudolph *et al*. (2007)
AU1090	*tnaA::*Tn*10 dnaA46*(ts) Δ*recG263::kan*	AU1068 × P1.N3793 to Km^r^
AU1106	Δ*recG263::kan*	N1141 × P1.N3793 to Km^r^
MG1655 and derivatives^b^		
MG1655	F^–^*rph-1*	Bachmann (1996)
APS345	*att*Tn*7::lacO240::kan zdd/e::tetO240::gen*	Rudolph *et al*. (2007)
N4280	*uvrA277::*Tn*10*	Rudolph *et al*. (2007)
N4560	Δ*recG265::cat*	Mahdi *et al*. (2006)
N5187	*sfiA100::kan pyrD*	MG1655 × P1.JJC396 to Km^r^
N5209	*sfiA11*	N5187 × P1.JJC395 to Pyr^+^ (Km^s^)
N5225	*sfiA11*Δ*recG265::cat*	N5209 × P1.N4452 to Cm^r^
N5466	Δ*ruvC::cat*	Mahdi *et al*. (2006)
N5500	*priA300*	Jaktaji and Lloyd (2003)
N5511	*priA300*Δ*recG263::kan*	N5500 × P1.N3793 to Km^r^
RCe29	*priA300*Δ*recG265::cat*	N5511 × P1.N4560 to Cm^r^ (Km^s^)
RCe72	*att*Tn*7::lacO240::kan zdd/e::tetO240::gen*Δ*recG265::cat*	APS345 × P1.N4560 to Cm^r^
RCe78	*att*Tn*7::lacO240::kan zdd/e::tetO240::gen*Δ*recG265::cat thr-43::*Tn*10*	RCe72 × BW6164 to Tc^r^
RCe79	*dnaC7*	Rudolph *et al*. (2007)
RCe93	*dnaC7 att*Tn*7::lacO240::kan zdd/e::tetO240::gen*	Rudolph *et al*. (2007)
RCe94	*att*Tn*7::lacO240::kan zdd/e::tetO240::gen*Δ*recG265::cat dnaC7*	RCe78 × P1.NY171 to Thr^+^
RCe97	*att*Tn*7::lacO240::kan zdd/e::tetO240::gen*Δ*ruvC::cat*	APS345 × P1.N5466 to Cm^r^
RCe107	*priA300 att*Tn*7::lacO240::kan*	N5500 × P1.APS345 to Km^r^
RCe108	*priA300 att*Tn*7::lacO240::kan zdd/e::tetO240::gen*	RCe107 × P1.APS345 to Gen^r^
RCe109	*priA300 att*Tn*7::lacO240::kan zdd/e::tetO240::gen*Δ*recG265::cat*	RCe108 × P1.N4560 to Cm^r^
RCe111	*dnaC7*Δ*recG265::cat*	RCe79 × P1.N4560 to Cm^r^
RCe197	*att*Tn*7::lacO240::kan zdd/e::tetO240::gen tnaA::*Tn*10 dnaA46*	APS345 × P1.RUC663 to Tc^r^
RCe198	*att*Tn*7::lacO240::kan zdd/e::tetO240::gen*Δ*recG265::cat tnaA::*Tn*10 dnaA46*	RCe72 × P1.RUC663 to Tc^r^
RCe259	*priA300 att*Tn*7::lacO240::kan zdd/e::tetO240::gen* Δ*recG265::cat tnaA::*Tn10 *dnaA46*	RCe109 × P1.RUC663 to Tc^r^

a The abbreviations kan, cat and gen refer to insertions conferring resistance to kanamycin (Km^r^), chloramphenicol (Cm^r^) and gentamicin (Gen^r^) respectively. Tn*10* confers resistance to tetracycline (Tc^r^). Strains carrying *dnaA46* or *dnaC7* are temperature sensitive for growth. CGSC: Coli Genetic Stock Center, Yale University.

b Only the relevant additional genotype of the derivatives is shown.

### Media and general methods

Luria–Bertani (LB) broth and 56/2 salts media, and methods for monitoring cell growth, P1vir transduction and determining sensitivity to UV have been cited (McGlynn and Lloyd, 2000).

### Multiplication of cells surviving UV irradiation

To monitor recovery of cells surviving UV irradiation, cultures of the strains indicated were grown in LB broth to an A_650_ of 0.2. The cells were pelleted, UV-irradiated on the surface of LB agar and resuspended in the original, but filter-sterilized, supernatant and diluted 10 000-fold in conditioned medium, which was created by growing the wild type strain in fresh LB broth to an A_650_ of 0.2 with subsequent sterile filtration. The diluted cells were incubated in a 37°C shaking water bath and at each time point samples were removed, mixed with 2.5 ml of molten 0.6% top agar kept at 42°C and plated on LB agar. At later time points the samples were diluted a further 10- or 100-fold in conditioned medium before plating. Colonies were counted after incubation for 18–24 h at 37°C.

### Single cell analyses

Cells were grown in LB broth to an A_650_ of 0.2. Microscope slides were equipped with a Gene Frame® (ABgene) and filled with LB agar (0.8%). 2.5 μl of the culture was added on top, the samples irradiated, the gene frames sealed with a coverslip and the slides examined on a heated (sample temperature ∼35°C) microscope stage (INSTEC) with a BX-52 Olympus microscope equipped with a coolSNAP™HQ camera (Photometrics). Pictures were taken at 5 min intervals. Images were taken and analysed by MetaMorph 6.2 (Universal Imaging) and processed using MetaMorph and Adobe Photoshop CS4.

### Control of RecG expression

For constitutive expression, strains deleted for the chromosomal *recG* carried pAM208. For induced expression, they carried pDIM104 or pECR12. Strains carrying the appropriate plasmid were grown in LB broth supplemented with carbenicillin (40 µg ml^−1^) and UV irradiated on the surface of LB agar as described for fluorescence microscopy. The irradiated cells were resuspended in the original, but filter-sterilized, supernatant and incubation continued at 37°C. RecG was induced by adding arabinose to a final concentration of 0.2%. Microscope slides were prepared as described above except the LB agar was supplemented with carbenicillin and arabinose.

### Thymine dimer removal

Removal of thymine dimers was as described (Rudolph *et al*., 2007). Briefly, cells were grown in LB broth and UV irradiated as for fluorescence microscopy. Two millilitres of samples was removed, DNA extracted via phenol chloroform extraction as described (Rudolph *et al*., 2007) and the concentration adjusted with TE to 250 μg ml^−1^. Following denaturation by boiling, 500 ng samples were transferred to a Zeta-Probe GT Membrane via dot blot. DNA was baked on the membrane for 2 h at 80°C, probed with mouse anti-CPD antibody (Sigma), diluted 1:1000 and complexes detected with sheep anti-mouse, alkaline phosphatase-conjugated secondary antibody (Sigma), diluted 1:10 000 as described for detection of BrdU. Signal was developed using an AttoPhos® AP Fluorescent Substrate System (Promega), measured using a STORM scanner system (Molecular Dynamics) in fluorescence mode (450 nm excitation, 520 nm emission) and quantified with ImageQuant 5.2.

### Southern analysis of the origin to terminus ratio

The origin to terminus ratio was determined in synchronized cells by Southern analysis of DNA extracted at various times after release from synchrony. The extracted DNA was probed with PCR fragments matching sequences at *oriC* or in the termination region (Rudolph *et al*., 2007).

### Fluorescence Microscopy

Fluorescence microscopy was as described (Rudolph *et al*., 2007). Briefly, cells were grown to an A_650_ of 0.2 in LB broth supplemented with 0.5 mM IPTG and 40 ng ml^−1^ anhydrotetracycline. A 1 ml sample was removed and expression induced in this sample at high levels by adding arabinose to 0.2%. The rest of the cells were pelleted, UV-irradiated on the surface of LB agar and resuspended in the original, but filter-sterilized, supernatant to continue incubation. One millilitre of samples was removed every 30 min and expression induced with arabinose for 30 min. A small sample of cells was transferred to a thin 1% LB agarose layer on microscopic slides and visualized with a BX-52 Olympus microscope equipped with a coolSNAP™HQ camera (Photometrics). eCFP and eYFP foci were visualized using the JP4-CFP-YFP filterset 86002v2 (Chroma). Images were taken and analysed by MetaMorph 6.2 (Universal Imaging) and processed using MetaMorph and Adobe Photoshop CS4.

### Measurement of DNA synthesis

Measurement of DNA synthesis was as described (Rudolph *et al*., 2007). Briefly, cultures were grown with vigorous shaking at 30°C in Davis medium supplemented with 1% casamino acids and 5 μg ml^−1^ thymidine. At an A_650_ of 0.2, the cells were filtered onto 0.22 μm cellulose acetate (Corning) and irradiated directly on the filter, or mock irradiated, before resuspending in the filtrate. [^3^H]thymidine (specific activity 80.0 Ci mmol^−1^, Amersham) was added to 2 μCi ml^−1^ before continuing incubation as indicated. Twenty microlitres of samples was taken at intervals, applied to 2.5 cm^2^ filters (Whatman 3MM) and immediately immersed in ice-cold 5% trichloroacetic acid for a minimum of 30 min. Filters were washed in three changes of fresh trichloroacetic acid and two of ethanol, dried and the bound radioactivity counted by liquid scintillation.
