# Supplementary material for: Pathological replication in cells lacking RecG DNA translocase
Source: Mol Microbiol. 2009 Jul 1;73(3):352–66. doi: 10.1111/j.1365-2958.2009.06773.x (PMC2764109; doi:10.1111/j.1365-2958.2009.06773.x)
Supplement: Supplementary file 3 [file mmi0073-0352-SD3.pdf]

Pathological replication in cells lacking RecG DNA translocase – Supporting information

*Christian J. Rudolph, Amy L. Upton, Lynda Harris and Robert G. Lloyd**

Institute of Genetics, University of Nottingham, Queen's Medical Centre,
Nottingham, NG7 2UH, United Kingdom

*for correspondence

E-mail bob.lloyd@nottingham.ac.uk

Tel (+44) 115 823 03 03
Fax (+44) 115 823 03 38

Christian J. Rudolph, Amy L. Upton, Lynda Harris and Robert G. Lloyd

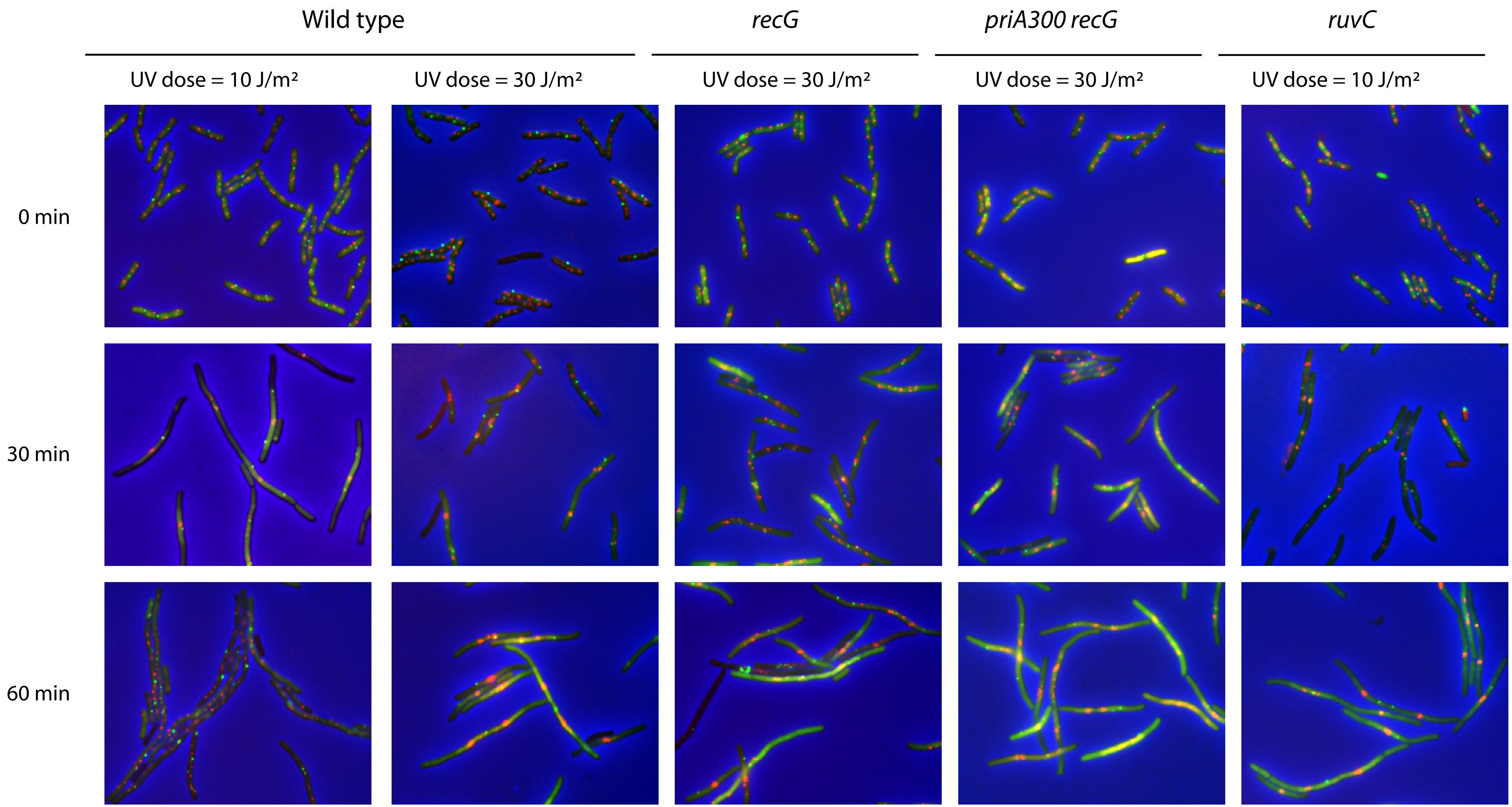

Fig. S1. Replication and segregation of origin and terminus areas in UV-irradiated *recG*, *priA300 recG* and *ruvC* cells. Fluorescence microscopy of *recG* (RCe72), *priA300 recG* (RCe109) and *ruvC* (RCe97) cells showing replication of origin (red foci) and terminus (green foci) areas of the chromosome (combined phase contrast and fluorescence images are shown). Data for wild type (APS345) were reproduced for comparison from (Rudolph *et al.*, 2007) with permission from Cold Spring Harbor Laboratory Press.

Christian J. Rudolph, Amy L. Upton, Lynda Harris and Robert G. Lloyd

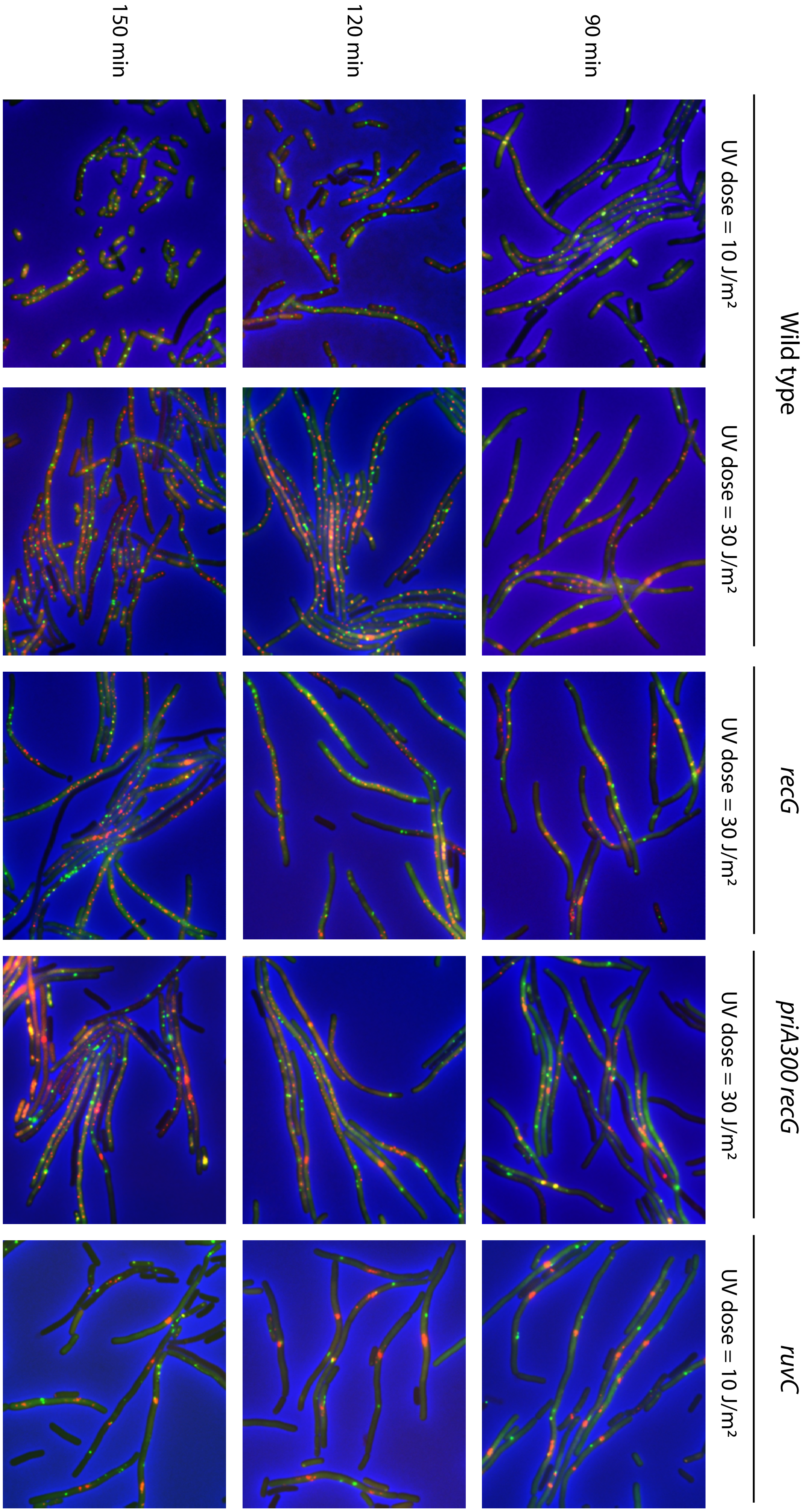

Christian J. Rudolph, Amy L. Upton, Lynda Harris and Robert G. Lloyd

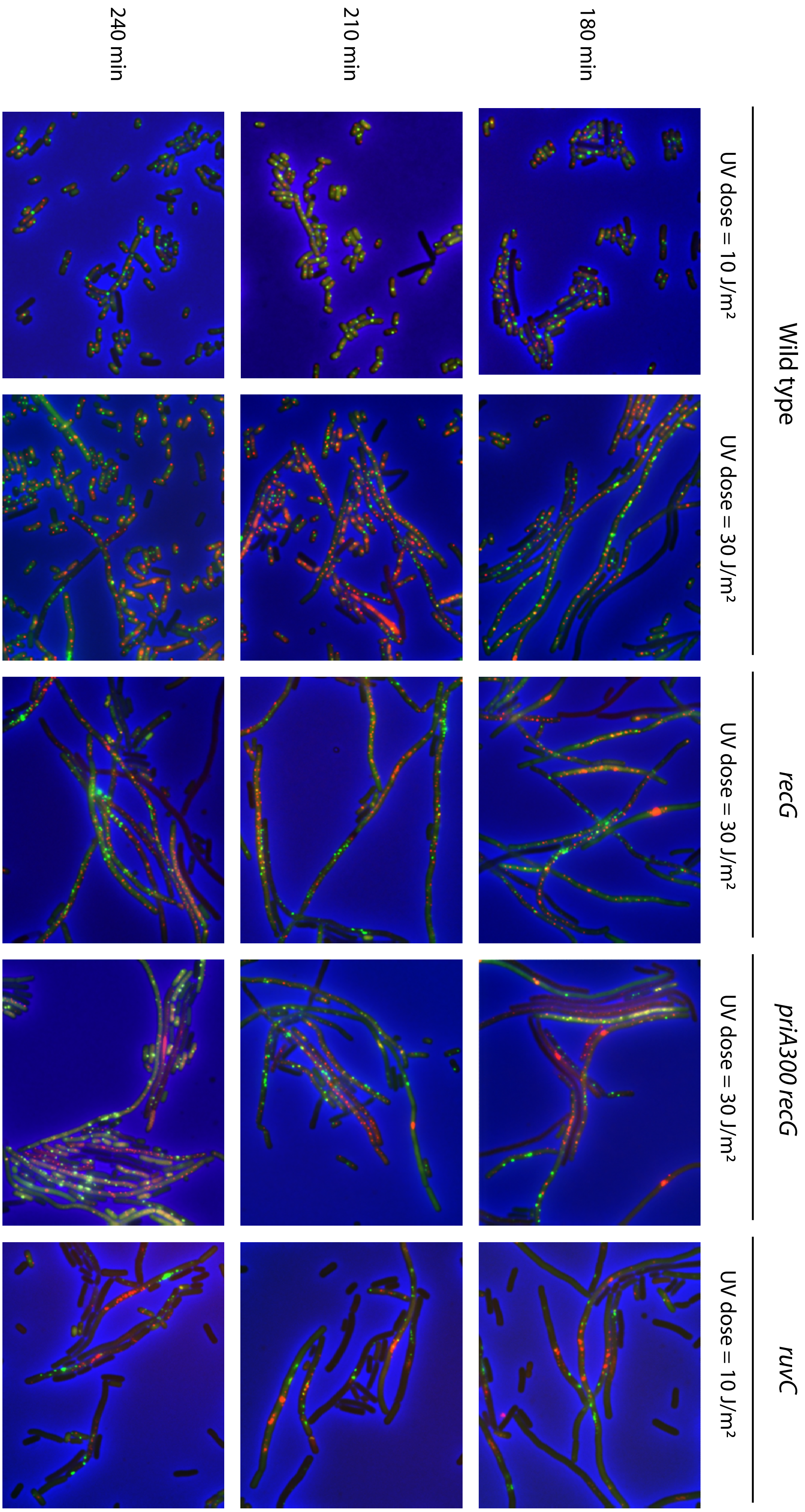

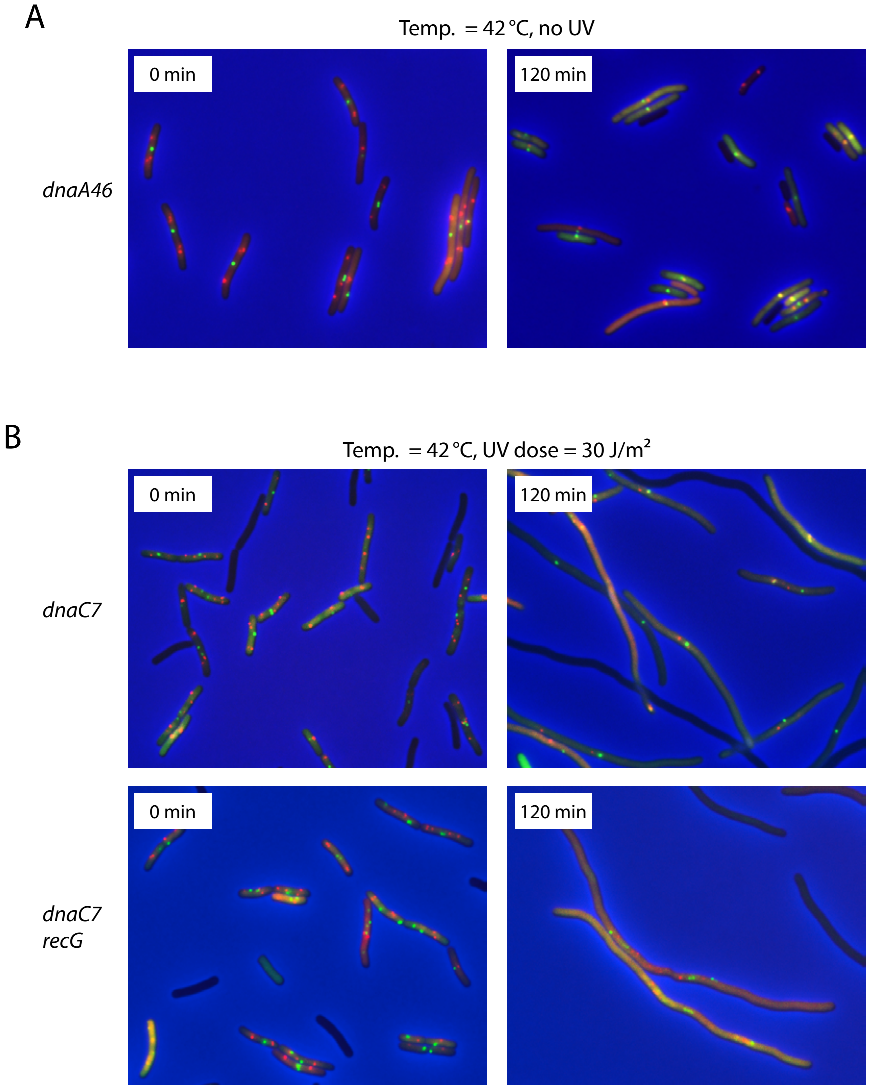

Fig. S2. Replication and segregation of origin and terminus areas in temperature sensitive *dnaA* and *dnaC* strains. (**A**) *dnaA46* cells shifted to restrictive temperature show a decrease in the ratio of origin (red) and terminus (green) foci, indicating that chromosomes can be segregated after completion of chromosomal replication (combined phase contrast and fluorescence images are shown). Cells were grown at permissive temperature and shifted to 42 °C after mock UV irradiation. The strain used was RCe197 *(dnaA46)*. (**B**) UV-induced synthesis is dependent on the helicase loader DnaC. No UV-induced increase in origin (red) and terminus foci (green) is observed in *dnaC7* strains at restrictive temperature (combined phase contrast and fluorescence images are shown). Cells were grown at permissive temperature prior to UV irradiation and shifted to 42 °C directly after UV. The strain used was RCe94 *(dnaC7 recG)*. Data for RCe93 *(dnaC7)* were reproduced for comparison from (Rudolph *et al.*, 2007) with permission from Cold Spring Harbor Laboratory Press.

Supplemental Movies

Movie 1. Time-lapse microscopy showing growth of wild type strain MG1655 cells after irradiation with 10 J/m^2 UV and incubated on the surface of LB agar.

Movie 2. Time-lapse microscopy showing growth of *recG* strain N4560 cells after irradiation with 10 J/m^2 UV and incubated on the surface of LB agar.

Supplemental References

Rudolph, C. J., A. L. Upton & R. G. Lloyd, (2007) Replication fork stalling and cell cycle arrest in UV-irradiated *Escherichia coli*. *Genes Dev* **21**: 668 – 681.
